# Synchronous Endobronchial Carcinoid Tumor and Adenocarcinoma of the Lung: A Case Report and Review of the Literature

**DOI:** 10.7759/cureus.15977

**Published:** 2021-06-27

**Authors:** Nada Hajjaj, Mohammad Abdulelah, Nada M Alsharif, Elias Shamieh, Husam Bader

**Affiliations:** 1 Department of Internal Medicine, University of Jordan, Amman, JOR; 2 Department of Internal Medicine, Presbyterian Medical Center, Albuquerque, USA

**Keywords:** adenocarcinoma, carcinoid tumor, multiple lung tumors, synchronous lung tumors, metachronous lung tumors

## Abstract

Diagnosis of synchronous multiple primary lung cancers (SMPLCs) is a challenge as multiple lesions on chest CT imaging can be misdiagnosed as more common entities such as metastatic disease or infections. The possibility of multiple primary lung cancers should always be considered. Accurate diagnosis can significantly change the management and prognosis. We report a case of a 57-year-old woman, an ex-smoker with chronic obstructive pulmonary disease (COPD), who was found to have synchronous endobronchial carcinoid tumor and adenocarcinoma of the lung. The association of carcinoid tumors and adenocarcinoma of the lung has been infrequently reported, with only a handful of cases published to date. Early diagnosis of resectable tumors can improve survival in patients with SMPLCs.

## Introduction

Lung cancer is the most common cause of cancer-related death for both sexes in the United States [1]. The differentiation between multiple primary lung tumors and metastatic lesions is of utmost importance as both management and prognosis can vary widely [2].

Multiple primary tumors can be synchronous or metachronous. Synchronous tumors are two or more tumors arising at the same time but with separate and distinct foci. The tumors may have similar or different histology [3]. Metachronous tumors are two or more tumors arising at different times in the same patient; most reported cases have different histology. If the tumors have similar histology, a two-year tumor-free period is required to diagnose metachronous tumor [4].

Among patients with lung cancer, the incidence of multiple primary lung tumors (both synchronous and metachronous) can vary from less than 1% to 16% [3]. Synchronous tumors are much less common than metachronous tumors, with an incidence of 0.5% to 2% in patients with lung cancer [5].

We report a case of a 57-year-old woman, an ex-smoker with chronic obstructive pulmonary disease (COPD), who was found to have synchronous endobronchial carcinoid tumor and adenocarcinoma.

## Case presentation

A 57-year-old female with mild COPD who presented to her primary care physician for fatigue and unintentional 40 lbs weight loss over the past year. Initial work-up noted a possible right hilar mass for which the patient was referred to the pulmonary clinic.

The patient’s known medical problems included mild COPD (per GOLD criteria for severity of airflow obstruction), well-controlled hypertension, hypothyroidism, obesity with BMI of 33, anxiety and a 15-20 pack-year smoking history. No pertinent family history of cancer. Upon interview, patient complained of a few weeks’ worth of chills and night sweats. She denied fever, shortness of breath, chest pain or hemoptysis. No recent travel or known sick contacts. Physical examination revealed a well-nourished woman with no acute distress. Respiratory auscultation with bilateral air entry and no additional sounds. She exhibited no chest tenderness. Her complete blood count and complete metabolic panel were normal.

**Figure 1:**
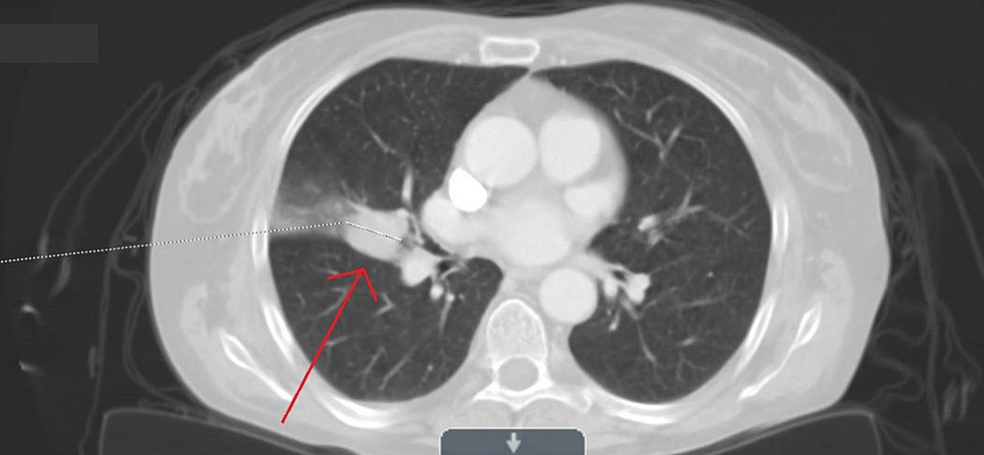
Chest CT showing a round 1.7 cm pulmonary nodule in the right perihilar region with adjunct ground-glass opacity. CT: computed tomography.

Initial chest radiograph revealed a possible right hilar mass. This was followed by a chest CT (Figure 1) which showed “a round 1.7 centimetre (cm) pulmonary nodule in the right perihilar region containing internal macroscopic fat density with suspected airway occlusion“. The nodule was suspected to either represent a pulmonary hamartoma or an endobronchial mass. Given the patient's weight loss and smoking history, the evaluation with bronchoscopy and biopsy was recommended.

**Figure 2:**
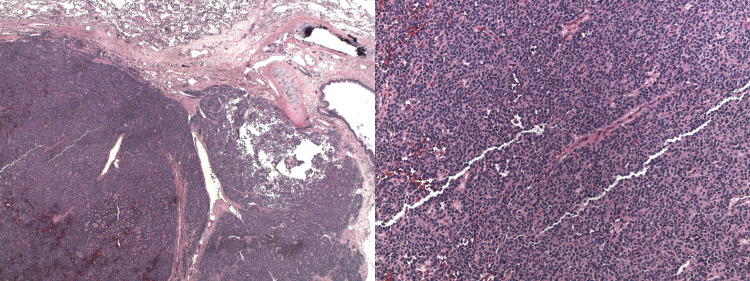
Biopsy results showing carcinoid tumor.

Bronchoscopy revealed an endobronchial lesion in the lateral segment of the right middle lobe and 14 biopsies were taken. Pathology reported differentiated neuroendocrine (carcinoid) tumor, grade 1, mitotic rate of <1%, and Ki-67 fraction of 2.1% (Figure 2). Alveolar parenchyma showed no specific abnormality. Bronchoscopy was followed by an Octreotide scan which showed only minimal activity associated with the right mid lung nodule, indicating a somatostatin receptor-positive neuroendocrine tumor.

A positron emission tomography (PET) scan was done and noted a 0.87 cm hypermetabolic nodule in the right middle lobe. Given the timeframe of appearance of this nodule, differential diagnosis favoured post-inflammatory changes or post-procedural reactive changes. There was no appreciated lymph node involvement on PET scan.

**Figure 3:**
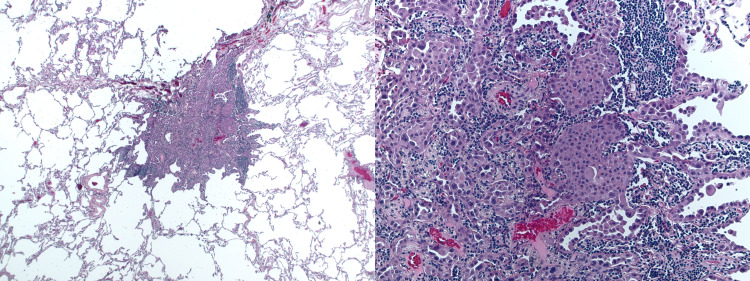
Pathology of the second mass showing adenocarcinoma of the lung.

Subsequently, lobectomy was performed by the cardiothoracic surgeon. Post-operative pathology showed an unexpected two unrelated primary tumors; The previously diagnosed differentiated neuroendocrine tumor and a minimally invasive non-mucinous adenocarcinoma in the right middle lobe (Figure 3). Interestingly, both tumors were stage 1 without any lympho-vascular or adjacent structure invasion. Parenchymal margins were negative.

Given the favorable staging and negative margins, lobectomy was felt to be therapeutic by the oncology team, and no adjuvant therapy was recommended. Surveillance protocol was done per National Comprehensive Cancer Network (NCCN) guidelines with CT chest every six months for the following two years. To date, patient remains disease-free with no evidence of recurrent malignancy in the lungs.

## Discussion

Accurate staging of lung cancer is important for directing treatment and prognosis. A retrospective study at an academic center reviewed 83 patients who had undergone surgical resection of primary lung cancer with the intention of cure. Of these cancers, 20.5% had pathologically confirmed synchronous multiple primary lung cancers [6].

The scientific data addressing synchronous multiple primary lung cancers (SMPLCs) is not extensive. In 1924, Beyrenther first described them while doing post-mortem studies, the cancers he mentioned were small cell carcinoma along with adenocarcinoma of the lung [7]. Subsequently, few cases were reported describing synchronous squamous cell carcinoma with small cell carcinoma of the lung or synchronous squamous cell with squamous cell carcinoma [8].

Differentiation between synchronous and metachronous lung cancers is a challenging task. In 1975, Martini and Melamed proposed the need for two histologically distinct tumors to diagnose MSPLCs, except in cases where the tumor occurs in a different segment, lobe, or lung [9]. Subsequently, immunohistochemical staining [5] and other factors were added to diagnose tumors with similar histology.

A retrospective analysis noted smoking to be an independent risk factor for the development of SMPLCs by inducing multifocal lung damage [10]. A phenomenon known as “field cancerization” is used when a certain carcinogen puts an entire field at risk of cancer [11]. Therefore, our patient’s heavy smoking history (15-20 pack-year) may have contributed to the development of synchronous primary lung tumors.

There may be a misconception that the presence of multiple tumors indicates inevitable unresectability and is an indication for non-surgical first-line treatment. Synchronous primary tumors and primary lung tumors with intrapulmonary metastasis can be amenable for resection per the new recommendations of the 8th edition of the TNM classification of lung cancer [12]. In a metachronous lung tumor, the clinical stage of the second tumor determines if surgery is feasible or not, whereas, in synchronous lung tumors, each tumor should be staged and managed separately [13]. In our patient, due to favorable staging of both primary tumors, only surgical resection was done.

The association of lung adenocarcinoma and bronchial carcinoid is extremely rare with the first report published in 1966 by Roberts and Cumming [14]. Since 1966, only seven other cases were found describing synchronous endobronchial carcinoid and adenocarcinoma via a search conducted on PubMed, Google scholar, and Mednar using the keywords: “Synchronous”, “lung”, “adenocarcinoma” and “carcinoid” (Table 1). One study reported a case of triple synchronous lung cancer [15], and another study reported five synchronous tumors in one patient within seven years [16].

**Table 1:** Case reports. Summary of seven published cases documenting synchronous lung tumors with pathology showing neuroendocrine and adenocarcinoma features. The databases included in the search were PubMed, Google scholar, and Mednar.

Case Report	Age	sex	Smoking history	Stage at the time of diagnosis	Management	Follow up
Yano et al. (2002) [17] (Original article in Japanese)	58	Female	-	-	Right middle and lower bilobectomy and mediastinal lymph node dissection	-
Nagamstu et al. (2012) [18]	67	Female	73.5 pack-year smoking history	Cancer-in-cancer. Stage IA (T1aN0M0)	Right upper lobectomy with mediastinal lymph node dissection	-
Saladi et al. (2018) [2]	68	Male	25 pack-year smoking history.	Stage IV adenocarcinoma with pleural metastasis	Palliative chemotherapy	-
Jung-Legg et al. (1986) [15]	49	Male	60 pack-year smoking history.	-	Right upper and middle lobectomy, adjunctive chemotherapy, and prophylactic irradiation to the brain.	-
Flynn et al. (2004) [16]	63	Male	Previous smoker.	-	Right upper lobectomy (for latest presentation with synchronous triple tumors)	-
Mikhail et al. (2020) [19]	65	Female	50 pack-year smoking history.	-	-	-
Sen and Borczuk (1998) [20]	60	Female	45 pack-year smoking history.	Stage II adenocarcinoma	Left lower lobe lobectomy	A follow-up chest (CT scan 1 year later was negative for recurrent tumor or lymphadenopathy)

Four of the seven patients underwent surgical lobectomy with intent to treat, one underwent lobectomy with adjuvant chemotherapy and prophylactic radiation to the brain, one underwent palliative therapy due to advanced stage and the last had no described treatment plan. Follow-up was mentioned in only one of the seven cases where the patient had undergone lobectomy for stage II adenocarcinoma with synchronous neuroendocrine tumor. The patient remained in remission for the time of follow-up. The outcome mirrors that of the case we presented.

The median age of presentation was 63 years, with no gender predominance. The most striking feature between the cases published describing synchronous neuroendocrine and adenocarcinoma lung cancer was the extensive smoking history (median of 50 pack-year). This can be explained by the previously mentioned phenomenon of “field cancerization”.

## Conclusions

SMPLCs may present as multiple synchronous indeterminate lung nodules on chest CT. Diagnosis is challenging as this rare entity is often overlooked and excluded in the initial differential diagnoses in favor of more common etiologies. A high level of suspicion in patients with a smoking history coupled with accurate staging is essential. A multidisciplinary approach is of merit due to the rare and evolving nature of this entity. Lobectomy can be curative in the early stages.
